# An overview of the sandfly fauna (Diptera: Psychodidae) followed by the detection of *Leishmania* DNA and blood meal identification in the state of Acre, Amazonian Brazil

**DOI:** 10.1590/0074-02760200157

**Published:** 2020-11-16

**Authors:** Thais de Araujo-Pereira, Daniela de Pita-Pereira, Sandylere Moreira Baia-Gomes, Mariana Boité, Franklin Silva, Israel de Souza Pinto, Raimundo Leoberto Torres de Sousa, Andressa Fuzari, Cristian de Souza, Reginaldo Brazil, Constança Britto

**Affiliations:** 1 Fundação Oswaldo Cruz-Fiocruz, Instituto Oswaldo Cruz, Laboratório de Biologia Molecular e Doenças Endêmicas, Rio de Janeiro, RJ, Brasil; 2 Fundação Oswaldo Cruz-Fiocruz, Instituto Oswaldo Cruz, Laboratório de Pesquisa em Leishmanioses, Rio de Janeiro, RJ, Brasil; 3 Fundação Oswaldo Cruz-Fiocruz, Centro De Desenvolvimento Tecnológico em Saúde, Laboratório de Modelagem de Sistemas Biológicos, Rio de Janeiro, RJ, Brasil; 4 Instituto Federal de Educação, Ciência e Tecnologia do Pará, Itaituba, PA, Brasil; 5 Fundação Oswaldo Cruz-Fiocruz, Instituto Oswaldo Cruz, Laboratório de Doenças Parasitárias, Rio de Janeiro, RJ, Brasil

**Keywords:** cutaneous leishmaniasis, sandflies, *Leishmania* DNA-detection, blood meal analysis, vector-borne disease

## Abstract

**BACKGROUND:**

In Acre state, Brazil, the dissemination of cutaneous leishmaniasis has increased in recent years, with limited knowledge of the potential *Leishmania* spp. vectors involved.

**OBJECTIVES:**

Here, data concerning the sandfly fauna of Brasiléia municipality, *Leishmania* DNA-detection rates and the identification of blood meal sources of insects captured in 2013-2015 are presented.

**METHODS:**

Parasite detection in female sandflies was performed individually by multiplex polymerase chain reaction (PCR) (*Leishmania* kDNA/sandfly *cacophony-*gene), with the identification of *Leishmania* spp. by hsp70-PCR and sequencing. The identification of blood gut-content from fed females was performed by *cyt b*-PCR and sequencing.

**FINDINGS:**

A total of 4,473 sandflies were captured. A subgroup of 864 non-blood-fed females evaluated for the presence of *Leishmania* DNA showed 2.9% positivity for *Leishmania (Viannia) braziliensis* and *L*. *(V.) guyanensis*. The identification of blood meal sources was performed in 96 blood-fed females, allowing the identification of 13 vertebrate species. In nine/96 fed females, DNA from *L*. *(V.) shawi*, *L. (V.) guyanensis*, *L*. *(V.) braziliensis* and *Endotrypanum* sp. was detected.

**MAIN CONCLUSIONS:**

In *Brumptomyia* sp. and *Evandromyia termitophila*, the first report of *Leishmania* DNA-detection is provided in Acre; *Nyssomyia shawi* is implicated as potential vector of *L*. *(V.) braziliensis* and *L*. *(V.) guyanensis* for the first time in Brazil.

Leishmaniasis is a public health problem in 98 countries distributed across four continents (the Americas, Europe, Africa and Asia), with compulsory notification in 32 countries, including Brazil. The World Health Organization (WHO) estimates that 350 million people are at risk of contracting the infection, accounting for approximately 2 million new cases of the different clinical forms per year. Leishmaniasis is considered one of the six most important infectious diseases due to its high detection coefficient and capacity to produce deformities.^1^

Cutaneous leishmaniasis (CL) is a zoonosis present in all Brazilian states^2^ that is caused by protozoa of the *Leishmania* genus (order Kinetoplastida, family Trypanosomatidae), which are transmitted by hematophagous sandflies of the subfamily Phlebotominae (family Psychodidae). It can affect humans when they accidentally participate in the natural cycle of maintenance of the disease because of activities that require their entry into forest environments or even due to the proximity of houses close to the edge of forests or even within them.^1^ CL is associated with a variety of dermotropic *Leishmania* species, with most of the diversity of parasites being found in the Amazon Region. The transmission of the etiological agents involves different sandfly species in specific associations with the parasites and their hosts, constituting the links of several transmission cycles that occur throughout the Brazilian territory.^3^ ^,^ ^4^

A total of 139,003 CL cases were registered in Brazil from 2012 to 2018, 46% of which were reported only in the North Region, with predominance in the states of Pará, Amazonas, Rondônia and Acre.^2^ In the state of Acre, located southwest of the Amazon, CL is endemic with high rates of incidence and prevalence.^5^ ^-^ ^7^ ^)^ Acre is geographically divided into two mesoregions (Vale do Juruá and Vale do Acre) and five microregions (Cruzeiro do Sul, Tarauacá, Sena Madureira, Rio Branco and Brasiléia). Through the analysis of CL cases in these microregions from 2012 to 2018, Rio Branco (2,652), Brasiléia (2,025) and Sena Madureira (1,142) showed the greatest numbers of reported cases, considering the total of 7,524 notifications throughout the state of Acre.^2^ ^)^ There are no records of human cases of visceral leishmaniasis (VL) in Acre; nevertheless, there is a need for constant monitoring of the phlebotomine fauna and domestic animals, particularly dogs, since the occurrence of *Lutzomyia longipalpis*, the main vector of *Leishmania (Leishmania) infantum* in Brazil, has been recorded in the municipality of Assis Brasil (Acre) and in Bolivia and Peru.^8^

Brasiléia is located on the border with Cobija municipality (District of Pando - Bolivia); are highly dependent on commercial activities and represent areas of high endemicity for CL, with great circulation of people between these cities. The present study aims to describe the sandfly fauna found in the municipality of Brasiléia and to evaluate the *Leishmania* DNA-detection index, followed by the molecular characterisation of the parasite species and, in parallel, the identification of blood meal sources in the captured insects. With the data obtained in this study, it will be possible to reveal new elements for better understanding the CL transmission cycle in the study area, which provides an attractive scenario for leishmaniasis research due to the scarcity of data in the region.

## MATERIALS AND METHODS

*Study area -* Brasiléia municipality is located in the south of Acre state, Brazil (11°00′S 68°44′W) (Figure) and is characterised by a restricted urban area and an extensive rural zone in the Amazonian forest, covering 3,916,505 km² and an estimated population of 26,278 inhabitants.^9^ Brasiléia is located 237 km southern of Rio Branco, bordering the municipality of Cobija (District of Pando - Bolivia) and the Brazilian municipalities of Epitaciolândia, Assis Brasil, Sena Madureira and Xapuri. The vegetation is composed of dense and open tropical forest with predominance of palm trees; the presence of liana is common in the tropical rainforest.^10^ The region exhibits an equatorial tropical climate with temperatures varying between 22 and 33ºC and annual precipitation of approximately 1,900 mm, with higher rainfall intensity between the months of November to March and a dry period from May to August.

The survey was undertaken in Brasiléia municipality, in areas of current transmission of CL that were previously established according to human cases registered in recent years by the National Health Foundation (FUNASA) and the local Fernando Azevedo Correia Centre of Health. According to the human registered CL cases (2,025 from 2012-2018), most of them occurred in forested areas close to residences in rural areas. Brasiléia is characterised by human occupation associated with agricultural and extractive activities and intense deforestation. The increasing CL cases reported in the municipality suggests an adaptation of sandflies on the transmission of CL agents in the peridomicile environment situated very close to forest areas, where animal farming in proximity to houses is frequently observed. 

*Sandfly capture and morphological identification -* Sandflies were captured between September 2013 and January 2015 in seven areas situated in the rural zone (transversely positioned along the Federal BR 317 Trans Pacific Highway and identified as *Ramal* or *Ramais*, based on the distance in kilometres from the centre of Brasiléia) (Figure). The sites of capture were named by areas and *Ramais*, comprising areas of high-density forest (areas A1-R4, A3-R13, A4-R18, A5-R59) and peridomicile environment (areas A2-R5, A6-R69, A7-R74). The rural zone presents a dense forest in one side of the dirt road and most of the residences situated in the other side do not present basic services such as sanitation. The houses are surrounded by forest and the presence of several animals in the peridomicile can be observed, providing a favourable environment for the attraction of insect vectors and the occurrence of tropical diseases. 

**Figure:**
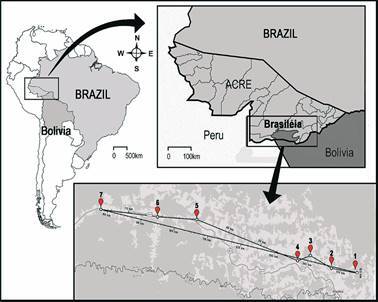
Municipality of Brasiléia, state of Acre, Brazil. The inset shows a satellite image indicating the distances between the seven study areas where sandflies were captured. Rural areas (located transversely along Trans Pacific Federal Highway BR 317): Area 1 - Kilometre 4, Ramal do Polo or Ramal 4 (10°56’43.43’’S, 68°42’13.91’’W); Area 2 - Kilometre 5, Ramal do Jarinal or Ramal 5 (10°56’6.30’’S, 068°46’4.57’’W); Area 3 - Kilometre 13, Ramal 13 (10°54’30.9’’S, 68°49’27.1’’W); Area 4 - Kilometre 18, Ramal 18 (10°55’20.8’’S, 68°51’29.1’’W); Area 5 - Kilometre 59, Ramal 59 (10°49’33,72’’S, 69°7’43.85’’W); Area 6 - Kilometre 69, Ramal 69 (10°49’8.84’’S, 069°14’3’’W); Area 7 - Kilometre 74, Ramal 74 (10°48’8.84’’S, 069°23’9,78’’W).

The selected areas were considered strategic for regular sandfly captures due to the high incidence of human CL cases in the neighbouring population. Captures were performed in private areas after receiving permission from the landowners. The sites of capture were georeferenced and the coordinates were obtained using a Global Positioning System (GPS) device. Sandfly captures were performed under a permanent license from the Biodiversity Authorisation and Information System (SISBIO) for the capture of zoological material (process 32669-4).

Sandflies were captured using light traps (HP model) placed at ground level (50-100 cm high) or at the top of trees (10-15 m high). In a first visit to recognise the areas, only ground level HP light traps were distributed in the peridomicile environments (areas A2-R5, A6-R69 and A7-R74) during 12 h (06:00 pm to 06:00 am). For the dense forest areas (A1-R4, A3-R13, A4-R18 and A5-R59), light traps were positioned during 24 h (from 06:00 pm to 06:00 pm of the following day), at the ground and at the top of trees in the forested environment near the houses. In addition, to determine phlebotomine anthropophily, a modified Shannon trap (model in “T”) with an attractive light was used from 06:00 pm to 09:00 pm under appropriate biosafety conditions [personal protective equipment (PPE)]. The strategy of captures is described in Table I.

**TABLE I:** Sandfly captures’ strategy from September 2013 to January 2015 in Brasiléia, Acre, Brazil

Capture	Area-Ramal	Number of HP light traps			Capture exposure (hours x days)	Sampling effort - HP traps (number of traps x exposure)	Shannon trap	Capture exposure (hours x days)	Sampling effort - Shannon (exposure x two persons)	Sampling effort (HP + Shannon)
		Ground	Top of trees	Days (hours)	Time (hours)	Time (hours)	Days (hours)	Time (hours)	Time (hours)	Time (hours)
1º - Dry season September/2013	A2-R5	5	-	2 (12 /night)	24	120	-	-	-	-
	A6-R69	2	-	1 (12 /night)	12	24	-	-	-	-
	A7-R74	3	-	1 (12 /night)	12	36	-	-	-	-
					Total	180				180
2º - Rainy season January/2014	A3-R13	2	2	4 (24 /day)	96	384	2 (3 /night)	6	12	
	A4-R18	2	2	4 (24 /day)	96	384	2 (3 /night)	6	12	
					Total	768			24	792
3º - Dry season July/2014	A3-R13	2	2	4 (24 /day)	96	384	2 (3 /night)	6	12	
	A4-R18	2	2	4 (24 /day)	96	384	2 (3 /night)	6	12	
	A1-R4	4	4	2 (24 /day)	48	384	2 (3 /night)	6	12	
					Total	1,152			36	1,188
4º - Rainy season January/2015	A1-R4	4	4	2 (24 /day)	48	384	2 (3 /night)	6	12	
	A5-R59	4	4	4 (24 /day)	96	768	4 (3 /night)	12	24	
				Total	1,152			36	1,188

Insects were stored in 80% ethanol and transported to the laboratory for preliminary screening and classification by area and strategy of capture. Males and females were clarified and diaphanised according to Forattini^11^ and identified following the taxonomic key proposed by Galati.^12^ ^,^ ^13^ The abbreviation of species names followed Marcondes.^14^ The mounting of female specimens on glass slides was performed using only the last abdominal segments and head for the microscopic observation of the genital tract and head structures (palps and cibarium); the thorax and abdomen were used for *Leishmania* DNA-detection, characterisation of the parasite species and blood gut-content analysis. Following species identification, each specimen was placed in a 1.5 mL tube and stored at -20°C until DNA extraction.

*Polymerase chain reaction (PCR) assays for the detection of Leishmania DNA -* A sampling of sandfly females was random selected and individually analysed for the presence of *Leishmania* DNA. The genetic material of each specimen was extracted and submitted to a multiplex PCR assay following a previously established protocol.^15^ ^)^ The assay is able to simultaneously amplify the conserved minicircle region of *Leishmania* kDNA (primer A: 5’ GGC CCA CTA TAT TAC ACC AAC CCC 3’; primer B: 5’ GGG GTA GGG GCG TTC TGC GAA 3’)^16^ ^)^ and the IVS6 region of the cacophony gene of neotropical sandflies (5Llcac: 5´ GTG GCC GAA CAT AAT GTT AG 3´; 3Llcac: 5´ CCA CGA ACA AGT TCA ACA TC 3´);^17^ this sequence served as an internal control for enzyme activity (by checking for the presence of potential inhibitors in the insect samples) and DNA yield and purity.^15^ Rigorous procedures were followed to control potential contamination. Male sandflies were included as negative controls for the DNA extraction step and all instruments and working areas were previously decontaminated with a diluted chloride solution and ultraviolet light. DNA extracts from *Lu. longipalpis* females that were artificially infected by feeding on rabbit blood (inactivated at 56°C) containing 2 × 10^5^ *Leishmania (Viannia) braziliensis* promastigotes/mL and DNA purified from *L. (V.) braziliensis* promastigotes were used as positive controls.

*Identification of Leishmania species in kDNA-positive sandfly samples -* Parasite species identification in the positive sandfly samples was performed by PCR amplification of the *Leishmania hsp70* (target, hsp70C reverse: 5’ GGA CGA GAT CGA GCG CAT GGT 3’ and hsp70C forward: 5’ TCC TTC GAC GCC TCC TGG TTG 3’), generating a 234 bp fragment, as previously described.^18^ ^)^ The 234 bp products were subjected to a second round of amplification using the hsp70C reverse primer and a new forward primer 5’ (hsp70F2 GGA GAA CTA CGC GTA CTC GAT GAA G3’),^19^ which amplifies an internal 144 bp region of the *Leishmania hsp70* gene. The 144 bp products were cloned using pGEM^®^-T Easy Vector Systems (Promega, Madison, USA) following the manufacturer’s standard procedures. Recombinant plasmids containing the *Leishmania* DNA insert of each positive sandfly sample were subjected to DNA extraction using the commercial PureLink Quick Plasmid DNA Miniprep kit (Invitrogen, Carlsbad, USA), according to the manufacturer’s protocol and submitted to sequencing.

*Identification of blood meal sources in blood-fed sandfly females -* The identification of blood gut-content of engorged sandfly females was conducted by PCR-based analysis of the *cytochrome B* gene (*cyt b*), using primers previously described,^20^ followed by sequencing the 383 bp product. DNA extracted from male sandflies was used as negative control and DNA from *Lu*. *longipalpis* females previously fed on rabbit blood (inactivated at 56°C) serves as positive control for the amplification reactions.

*Sequencing -* For both the analyses of *Leishmania* DNA-detection and food source, purified DNA or amplified PCR products respectively were submitted to sequencing. Reactions were performed using Big Dye Terminator v.3.1 Cycle Sequencing (Applied Biosystems Foster City, USA) following the manufacturer’s specifications and assayed in the Sanger ABI 3730 Sequencing. Consensus sequences were obtained and edited using the software package Phred/Phrap/Consed version: 0.020425.c (University of Washington, Seattle, USA) and only those sequences with Phred values above 20 were used as contigs. The contigs were assembled and aligned in MEGA5 software^21^ and sequences were evaluated against NCBI *nr* database using BLASTn.^22^

*Data analysis -* The measures of diversity among the environments were obtained using the Shannon-Wiener’s (*H′*) diversity index, the Pielou’s equitability index (*J’*), Margalef ’s richness index and the Berger-Parker’s dominance index.^23^ Kruskal-Wallis was used to test the equality among the ecological descriptors in the different areas/environments. The male/female ratios were calculated by the chi-squared test (χ2) and the comparison of the insect’s averages by hour of capture in the rainy/dry seasons was performed by the Student’s *t* test. The analyses were carried out using the IBM SPSS 20 software, with a 5% level of significance.

*Ethics -* Access to genetic heritage has been registered into the National System for the Management of Genetic Heritage and Associated Traditional Knowledge (SisGen - A41DBDD).

## RESULTS

*Sandfly fauna -* A total of 4,473 sandflies were captured being the proportion of female (n = 2,297, 51.35%) higher than that of male (n = 2,176, 48.65%) [χ^2^ = 16.27; degrees of freedom (df) = 3; p < 0.05]. Sixty-one species were identified belonging to 15 genera: *Nyssomyia*, *Psychodopygus*, *Trichophoromyia*, *Lutzomyia*, *Evandromyia*, *Pintomyia*, *Psathyromyia*, *Bichromomyia*, *Pressatia*, *Brumptomyia*, *Viannamyia*, *Sciopemyia*, *Trichopygomyia*, *Micropygomyia* and *Migonemyia*. The *Nyssomyia* genus was the most frequently captured (41.12% of the total), followed by *Psychodopygus* (30.53%) and *Tricho-phoromyia* (11.62%) (Table II). The females identified as *Trichophoromyia* probably represent *Th*. *octavioi* and *Th*. *auraensis*, since up to now these species are not distinguishable. In the dense forest environment where the three methods of capture were applied (light traps near the ground and on the top of trees and Shannon trap), 4,389 specimens of 58 species were captured, whereas only 84 specimens belonging to 21 species were captured with light traps positioned near the ground in the peridomicile environment (Table II). *Ny. shawi* was the most frequent species (26.83% of the total) and occurred in all areas of forest environment, but it was not present in the captures in the peridomicile rural environment. The second more frequent was *Ps. davisi* (12.1%), followed by *Ps. carrerai* (6.6%), *Nyssomyia* sp. (5.9%), *Ny. whitmani* (4.7%) and *Th. octavioi* (4.6%). The other species together represented 39.3% of the total captured specimens. 

**TABLE II:** Abundance and distribution of sandflies captured from September 2013 to January 2015 in Brasiléia, Acre, Brazil

Genera	Species	Forest environment			TF		Peridomicile environment			TP	OP %
		A1R4	A3R13	A4R18	A5R59		A2R5	A6R69	A7R74		
*Nyssomyia*	*Ny. shawi*	823	89	34	254	1,200					26.83
	*Ny. whitmani*	143	28	8	31	210					0.46
	*Ny. antunesi*	72	74	19	28	193	6			6	4.45
	*Ny. umbratilis*	8	68	42	3	121					2.57
	*Ny. yuilli yuilli*	8	21	9	2	40					0.89
	*Nyssomyia sp.*	164	28	32	27	251	14			14	5.92
*Lutzomyia*	*Lu. sherlocki*	22	36	38	5	101					2.26
	*Lu. flabellata*	2			2	4					0.08
	*Lutzomyia sp.*	11	34	7	0	52					1.16
*Psychodopygus*	*Ps. davisi*	276	115	32	119	542					12.12
	*Ps. carrerai carrerai*	152	8	3	130	293					8.47
	*Ps. llanosmartinsi*	4			163	167					6.55
	*Ps. amazonensis*	27	12	13	46	98					2.19
	*Ps. hirsutus hirsutus*	32	13	2	39	86					1.92
	*Ps. paraensis*	6	10	10	25	51					1.14
	*Ps. geniculatus*	6	2		23	31					0.69
	*Ps. lainsoni*	21			6	27					0.60
	*Ps. chagasi*				11	11					0.24
	*Ps. claustrei*			1	2	3		1		1	0.08
	*Ps. bispinosus*				1	1					0.02
	*Ps. complexus*	1				1					0.02
	*Ps. dorlinsis*				1	1					0.02
	*Psychodopygus sp.*	29	8	5	12	54					1.21
*Trichopygomyia*	*Ty. dasypodogeton*	1	2	3		6					0.13
	*Trichopygomyia sp.*		3		1	4					0.08
*Trichophoromyia*	*Th. octavioi*	47	18	22	118	205					4.58
	*Th. auraensis*	47	3	8	54	112		1	4	5	2.62
	*Th. ubiquitalis*	5			2	7					0.15
	*Th. clitella*	1				1					0.02
	*Trichophoromyia sp.*	25	7	19	139	190					4.25
*Evandromyia*	*Ev. saulensis*	4	13	40	4	61	4	5		9	1.56
	*Ev. bacula*	4		2	2	8	1			1	0.08
	*Ev. infraspinosa*		1	3		4	1			1	0.11
	*Ev. termitophila*	1		3		4	1			1	0.11
	*Ev. monstruosa*		2	1		3					0.06
	*Ev. walkeri*			2		2					0.04
	*Ev. cortelezzii*							1		1	0.02
	*Ev. williamsi*		2			2					0.04
	*Evandromyia sp.*	1	7	9	1	18	8	4		12	0.67
*Pintomyia*	*Pi. serrana*	29	10	11	11	61			1	1	1.39
	*Pi. nevesi*	5	3	4	2	14	1	1		2	0.35
	*Pintomyia sp.*		2			2	1			1	0.06
*Bichromomyia*	*Bi. flaviscutellata*	1	1	20		22					0.51
	*Bi. olmeca nociva*		7	1		8	1			1	0.20
	*Bi. olmeca bicolor*				2	2					0.04
	*Bichromomyia sp.*		2	1		3					0.06
*Psathyromyia*	*Pa. aragaoi*	9	5	3	1	18	1		1	2	0.44
	*Pa. dendrophyla*	7		2	3	12	1			1	0.29
	*Pa. bigeniculata*		5			5					0.11
	*Pa. lutziana*		3	1		4	1			1	0.11
	*Pa. abonnenci*	2		1		3					0.06
	*Pa. campbelli*		2	1		3					0.06
	*Pa. scaffi*	1	1		1	3					0.06
	*Pa. abunaensis*	1				1					0.02
	*Pa. punctigeniculata*		1			1					0.02
	*Psathyromyia sp.*	4	7	3	5	19	1			1	0.44
*Viannamyia*	*Vi. furcata*	3	7	2		12					0.26
*Pressatia*	*Pr. choti*	3				3					0.06
	*Pr. duncanae*	2	1			3					0.06
	*Pr. calcarata*	1				1					0.02
	*Pr. triacantha*			1		1					0.02
	*Pressatia sp.*	7		1		8	2			2	0.22
*Brumptomyia*	*Br. galindoi*				1	1					0.02
	*Br. mangabeirai*			1		1					0.02
	*Br. avellari*						8			8	0.17
	*Brumptomyia sp.*						5			5	0.11
*Sciopemyia*	*Sc. sordellii*						2		3	5	0.11
	*Sc. servulolimai*		1	2		3					0.06
	*Sciopemyia sp.*		2	1		3	1			1	0.08
*Migonemyia*	*Mg. migonei*			1	1	2					0.04
	*Migonemyia sp.*			1		1					0.02
*Micropygomyia*	*Mi. longipennis*	3				3					0.06
	*Mi. trinidadensis*	1				1					0.02
	*Mi. micropyga*						1			1	0.02
	*Micropygomyia sp.*						1			1	0.02
Total		2,022	664	425	1,278	4,389^*a*^	62	13	9	84^*a*^	100

*a*: comparison for the abundance of sandflies in the forest and peridomicile environments. A: area; OP %: overall percentage; R: *Ramal* (defined by the distance in kilometres along the Trans Pacific Federal Highway BR 317); TF: total forest environment; TP: total peridomicile environment. Kruskal-Wallis test. *H* = 36.6, degrees of freedom (df) = 14, p = 0.001.

Captures were carried out in the dry and rainy seasons performing four capture campaigns (Table I). The first (2013 - dry season) was performed in areas A2-R5, A6-R69 and A7-R74, with a sampling effort of 180 h; 84 sandflies were trapped giving an average of 5.60 insects per trap. The second (2014 - rainy season) refers to areas A3-R13 and A4-R18, totalising 792 h; 394 specimens were captured with an average of 10.94 sandflies per trap. The third visit (2014 - dry season) contemplated areas A1-R4, A3-R13, A4-R18 with a sampling effort of 1,188 h; a total of 2,081 individuals was captured with an average of 38.53 insects per trap. The last capture (2015 - rainy season) was conducted in areas A1-R4 and A5-R59 with a sampling effort of 1,188 h; a total of 1,914 specimens was captured with an average of 35.44 sandflies per trap. All areas of the dense forest environment where the three methodologies of capture were applied (areas A1-R4, A3-R13, A4-R18 and A5-R59) presented identical sampling effort (792 h each). Of the total of 4,473 individuals, 51.6% (n = 2,308) were captured in the rainy season with a sampling effort of 1,980 h and an average of 1.16 individuals trapped per hour, whereas 48.4% of the total (n = 2,165) were captured in the dry season with a sampling effort of 1,368 h and an average of 1.58 individuals trapped per hour (p = 0,053, df = 4).

Relative to all studied areas of both forest and domicile environments, the most frequent species in the light traps located at the top of trees were *Ny. shawi* (27.5%, n = 499), *Ps*. *davisi* (13.1%, n = 239), *Ny*. *antunesi* (6.6%, n = 120), *Ps*. *llanosmartinsi* (6.5%, n = 118), *Ps*. *carrerai* (6%, n = 109) and *Ny*. *whitmani* (6%, n = 109). In the light traps near the ground, the most frequent species were *Th*. *octavioi* (13.5%, n = 178), *Ny*. *shawi* (11.8%, n = 156), *Trichophoromyia* sp. (11.3%, n = 149) and *Ps*. *davisi* (9.7%, n = 128). In Shannon traps, *Ny. shawi* (40.4%, n = 545) was the most frequently captured species, followed by *Ps. davisi* (13%, n = 175), *Ps. carrerai* (11.2%, n = 152), *Nyssomyia* sp. (7.1%, n = 96), *Ny. whitmani* (5.2%, n = 70) and *Lu. sherlocki* (3.8%, n = 52). The other specimens together (19%, n = 256) make up the total of 1,346 sandflies captured in Shannon. 

Considering only the dense forest environment with three methodologies of captures, the average numbers of sandflies per trap were 19.2, 28.3 and 84.1, respectively to HP light traps near the ground, on the top of trees and Shannon. In Shannon and light traps, *Ny*. *shawi* was the most captured species and area A1-R4 was the one with the highest number of specimens of the *Nyssomyia* genus. In the dense forest areas, 4,389 individuals were captured with the most abundant being *Ny. shawi*, *Ps. davisi*, *Ny. umbratilis*, *Ev. saulensis*, *Th. auraensis* and *Nyssomyia* sp. 

In the forest environment, area A1-R4, the diversity (Shannon index, *H*’) of species was 2.24 (S = 45) and equitability (*J’*) was 0.59, whereas in area A4-R18 the diversity was higher at 3.14 (S = 45) and equitability was also higher (*J’* = 0.82), demonstrating a more uniform pattern of species frequencies (Table III). Although both the areas present the same number of species, the number of individuals in area A4-R18 is much lower (A1-R4, n = 2,022 and A4-R18, n = 425) (Table II), despite the higher diversity of species in this area (*H* = 24.16, df = 14, p = 0,044). The other areas comprising the forest environment (A3-R13 and A5-R59) revealed diversity indexes of 2.90 (S = 42) and 2.61 (S = 38), and equitability (*J’*) 0.77 and 0.72, respectively. These results demonstrate a relatively minor balance for area A5-R59 compared to A3-R13 (*H* = 3.6, df = 14, p > 0.004). Species dominance, according to the Berger-Parker’s index, varied from 0.09 to 0.44 for both, forest and peridomicile environments. Only one area of the peridomicile environment (A2-R5) shows diversity index similar to the ones observed in the forest areas at 2.55 (S = 21), but with a lower number of species. Higher equitability values (*J*’) were observed in the peridomicile environment, varying between 0.83 to 0.87 (Table III). 

**TABLE III:** Ecological descriptors of sandflies captured from September 2013 to January 2015 in Brasiléia, Acre, Brazil

Ecological descriptors	Forest environment				Peridomicile environment		
	A1R4	A3R13	A4R18	A5R59	A2R5	A6R69	A7R74
Species richness (S’)	45	42	45	38	21	6	4
Shannon-Wiener index (*H’*)	2.24^*a*^	2.90^*b*^	3.14^*a*^	2.61^*b*^	2.55	1.51	1.21
Pielou equitability (*J’*)	0.59	0.77	0.82	0.72	0.83	0.84	0.87
Berger-Parker’s index (D’)	0.40	0.17	0.09	0.19	0.22	0.38	0.44
Dominant species per area	NS	PD	NU	NS	N	ES	TA

*a*: comparison for the diversity index (*H′*) between A1R4 and A4R18 areas Kruskal-Wallis test. *H* = 24.16, degrees of freedom (df) = 14, p = 0.044; *b*: comparison for the diversity index (*H′*) between A3R13 and A5R59 areas (Kruskal-Wallis test. *H* = 31.6, df = 14, p = 0.004). A: area; ES: *Evandromyia saulensis*; N: *Nyssomyia sp.*; NU: *Nyssomyia umbratilis*; PD: *Psychodopygus davisi*; R: *Ramal* (defined by the distance in kilometres along the Trans Pacific Federal Highway BR 317); NS: *Nyssomyia shawi*; TA: *Trichophoromyia auraensis*.

*Leishmania DNA-detection in non-blood-fed female sandflies* - A panel of 864 female sandflies without blood in their guts was analysed for the presence of *Leishmania* DNA (Table IV). Engorged sandflies were excluded from the detection assays due to the possibility of detecting occasional ingestion of blood from an infected source. In 25 individuals, positive results were observed by kDNA-PCR in agarose gels, corresponding to a 2.9% detection rate in the study area. The sandfly species of positive samples are indicated in Table V and these specimens were captured in all areas of forest environment and in only one peridomicile area (A2-R5). *Leishmania* DNA positive sandflies were captured in all four capture campaigns. Positive insects were not identified in area A6-R69, while the few specimens captured in area A7-R74 presented blood in their gut contents and were not subjected to the diagnosis of *Leishmania* spp. detection.

**TABLE IV:** Sandflies analysed for the presence of *Leishmania* DNA (non-blood-fed females)

Area	A1R4			A2R5	A3R13			A4R18			A5 R59			A6R69	Total
Type of trap	HP		Shannon	HP	HP		Shannon	HP		Shannon	HP		Shannon	HP	
	Top of tree	Ground		Ground	Ground	Top of tree		Top of tree	Ground		Top of tree	Ground		Ground	
Species															
*Nyssomyia shawi*	21	21	44		34	14	5	1	4	10	9	4	13		180
*Nyssomyia* sp.	19	28	5	11	16	6	4	3	9	19	6	9	9		144
*Trichophoromyia* sp.	6	11	1			4			14	2	15	22	3		78
*Psychodopygus davisi*	13	12	14		5	5	6	2	1	7	1		4		70
*Nyssomyia umbratilis*		4			6	3	3	20	6		1	1			44
*Psychodopygus carrerai*	3	3	18		1	1	1			1	2	4	8		42
*Evandromyia saulensis*		2			1	5	1		18	5				4	36
*Psychodopygus* sp.	7		6		2	3	3	1	1	3	4	3	1		34
*Psychodopygus amazonensis*	4		2		1	3	2		1	8	3		6		30
*Evandromyia* sp.				7	2	4			5	2				4	24
*Lutzomyia sherlocki*					1	1	3	1		14					20
*Nyssomyia antunesi*	1		2	1	7	2	2		1	3					19
*Nyssomyia yuilli yuilli*					11	1	1	4	1						18
*Psychodopygus hirsutus hirsutus*	7	1	3		1		1			2	2				17
*Psathyromyia* sp.	3			1	2				2		3	2			13
*Lutzomyia* sp.	2						7			3					12
*Nyssomyia whitmani*	5	6						1							12
*Psychodopygus paraensis*					1					10					11
*Pintomyia serrana*			1		1			2	1	3					8
*Psychodopygus lainsoni*	3	3	1												7
*Psychodopygus llanosmartinsi*											1		6		7
*Brumptomyia* sp.				5											5
*Pintomyia nevesi*				1		3			1						5
*Psychodopygus geniculatus*	1						1				3				5
*Pressatia* sp.		1		2				1							4
*Pintomyia* sp.				1		2									3
*Sciopemyia* sp.				1	1	1									3
*Evandromyia bacula*		1		1											2
*Psathyromyia dendrophyla*				1					1						2
*Trichopygomyia dasypodogeton*								2							2
*Bichromomyia flaviscutellata*										1					1
*Evandromyia monstruosa*										1					1
*Evandromyia termitophila*				1											1
*Psathyromyia bigeniculata*					1										1
*Psathyromyia lutziana*						1									1
*Psychodopygus complexus*			1												1
*Viannamyia furcata*								1							1
Total	95	93	98	33	94	59	40	39	66	94	50	45	50	8	864

R: *Ramal* (defined by the distance in kilometres along the Trans Pacific Federal Highway BR 317)*.*

The positivity for *Leishmania* DNA per sandfly species was distributed as follows: 0.69% in *Nyssomyia* sp. (one/144); 1.28% in *Trichophoromyia* sp. (one/78); 2.22% in *Ny. shawi* (four/180); 2.27% in *Ny. umbratilis* (one/44); 3.33% in *Ps. amazonensis* (one/30); 5.26% in *Ny. antunesi* (one/19); 5.88% in *Ps. hirsutus* (one/17); 5.88% in *Psychodopygus* sp. (two/34); 7.69% in *Psathyromyia* sp. (one/13); 8.33% in *Ny. whitmani* (one/12); 10% in *Ps. davisi* (seven/70); 20% in *Brumptomyia* sp. (one/5) and *Pi. nevesi* (one/5); 33.33% in *Pintomyia* sp. (one/3) and 100% detection found in *Ev. termitophila* (one/one) (Table V).

**TABLE V:** Female sandfly species with positive results for *Leishmania* spp. kDNA according to the area of capture, trap and the identified *Leishmania* species

Positive sandflies for *Leishmania* kDNA	Area-R/Trap	Parasite species (*hsp70*)	Accession number	BLAST analysis		Total
				E-value	Identity (%)	
*Brumptomyia* sp.	A2-R5/HP ground	*L. (V.) braziliensis*	GU368187.1	8e-62	99	1
*Evandromyia termitophila*	A2-R5/HP ground	*L. (V.) braziliensis*	GU368187.1	8e-67	100	1
*Nyssomyia antunesi*	A2-R5/HP ground	*L. (V.) braziliensis*	GU368187.1	8e-68	100	1
*Nyssomyia shawi*	A3-R13/HP tree	*L. (V.) braziliensis*	XM_0011566273.2	5e-69	100	1
	A1-R4/HP tree	*L. (V.) guyanensis*	FN395051.1	5e-69	100	1
	A1-R4/Shannon	*L. (V.) braziliensis*	GU368187.1	5e-69	100	1
		NI		-	-	1
*Nyssomyia* sp*.*	A5-R59/HP tree	NI		-	-	1
*Nyssomyia umbratilis*	A3-R13/HP tree	*L. (V.) braziliensis*	XM_001566273.2	2e-42	89	1
*Nyssomyia whitmani*	A1-R4/HP tree	NI		-	-	1
*Pintomyia nevesi*	A2-R5/HP ground	*L. (V.) braziliensis*	XM_001566273.2	2e-42	89	1
*Pintomyia* sp.	A2-R5/HP ground	NI		-	-	1
*Psathyromyia* sp.	A2-R5/HP ground	*L. (V.) braziliensis*	XM_001566273.2	1e-65	100	1
*Psychodopygus amazonensis*	A5-R59/Shannon	NI		-	-	1
*Psychodopygus davisi*	A3R13/HP ground	*L. (V.) braziliensis*	LN907833.1	3e-25	97	1
	A4-R18/Shannon	*L. (V.) braziliensis*	LN907845.1	5e-39	89	1
		*L. (V.) braziliensis*	XM_001566273.2	1e-65	100	1
		*L. (V.) braziliensis*	GU368187.1	3e-25	97	1
	A1-R4/Shannon	NI		-	-	2
	A5-R59/Shannon	NI		-	-	1
*Psychodopygus hirsutus hirsutus*	A4-R18/Shannon	*L. (V.) braziliensis*	LN907833.1	3e-25	97	1
*Psychodopygus* sp.	A1-R4/HP tree	NI		-	-	1
	A1-R4/Shannon	*L. (V.) guyanensis*	GU368213.1	5e-69	100	1
*Trichophoromyia* sp.	A1-R4/HP ground	*L. (V.) braziliensis*	LN907845.1	3e-25	97	1
Total						25

NI: not identified; R: *Ramal* (defined by the distance in kilometres along the Trans Pacific Federal Highway BR 317)*.*

The 25 positive samples were further analysed by *hsp70*-PCR and sequencing and in 16 of these samples (64%) it was possible to identify the *Leishmania* species. BLAST analysis revealed the presence of *L. (V.) brazili-ensis* DNA with 100% identity for the species *Ev. termitophila* (n = 1), *Ny. antunesi* (n = 1), *Ny. shawi* (n = 2), *Psathyromyia* sp. (n = 1) and *Ps. davisi* (n = 1). The other positive samples for *L. (V.) braziliensis* presented identity values between 89 and 99% and included females of *Brumptomyia* sp. (n = 1), *Ny. umbratilis* (n = 1), *Pi. nevesi* (n = 1), *Ps. davisi* (n = 3), *Ps. hirsutus hirsutus* (n = 1) and *Trichophoromyia* sp. (n = 1). In two species, *Ny. shawi* (n = 1) and *Psychodopygus* sp. (n = 1), it was possible to confirm infection by *L. (V.) guyanensis* with 100% identity (Table V).

*Blood meal and Leishmania DNA-detection in engorged female sandflies -* A panel of 96 female sandflies containing traces of blood meal observed under of a stereomicroscope was subjected to food source analysis after being tested for *Leishmania* DNA, with the aim of identifying potential reservoirs affecting the parasite transmission cycle(s) in the studied areas. Thirteen species of mammals were identified in 39 out of the 96 samples (40.6%): 41.02% (16/39) of these sequences corresponded to *Homo sapiens*; 10.26% (four/39) to each of *Dasypus novemcinctus* and *Tamandua tetradactyla*; 7.70% (three/39) to each of *Coendou prehensilis* and S*us scrofa*; 5.13% (two/39) to *Bos taurus*; and 2.56% (one/39) to each of *Agouti paca*, *Choloepus didactylus*, *Dasyprocta fuliginosa*, *Dasypus kappleri*, *Didelphis marsupialis*, *Marmosops noctivagus* and *Pecari tajacu* (Table VI). The remaining samples were left without feeding source identification due to the lack of similarity with those available sequences in the database or to the yield of low-quality sequences.

**TABLE VI:** Consolidated analysis of blood gut-contents in sandflies

Species	Blood meal (c*yt b*)	Accession number	BLAST analysis		Area-R/trap	Total
			E-value	Identity (%)		
*Bichromomyia olmeca nociva*	*Bos taurus*	KU891850.1	9e-35	86	A3-R13/HP ground	1
*Evandromyia saulensis*	*Homo sapiens*	DQ489515.1	6e-18	92	A3-R13/HP ground	1
	*Homo sapiens*	KP126161.1	2e-161	98	A4-R18/HP ground	1
	*Sus scrofa*	GQ338965.1	1e-42	91	A3-R13/HP ground	1
*Evandromyia* sp.	*Tamandua tetradactyla*	AF232019.1	2e-152	98	A2-R5/HP ground	1
*Lutzomyia sherlocki*	*Homo sapiens*	KM986625.1	9e-68	93	A1-R4/HP tree	1
	*Sus scrofa*	KJ652503.1	1e-17	95	A3-R13/HP ground	1
*Nyssomyia shawi*	*Homo sapiens*	KJ185819.1	2e-75	89	A4-R18/HP tree	1
	*Homo sapiens*	KC622257.1	5e-137	99	A1-R4/Shannon	1
*Nyssomyia* sp.	*Homo sapiens*	KT779190.1	3e-94	87	A2-R5/HP ground	2
	*Homo sapiens*	KR712115.1	6e-73	84		
Pintomyia nevesi	*Homo sapiens*	KT897693.1	8e-140	95	A4-R18/Shannon	1
*Pintomyia serrana*	*Agouti paca*	AY206572.1	2e-72	83	A5-R59/HP ground	1
	*Coendou prehensilis*	KC463874.1	4e-163	100	A4-R18/HP tree	1
	*Homo sapiens*	KP126161.1	8e-165	99	A1-R4/HP ground	1
*Psathyromyia aragaoi*	*Homo sapiens*	KP126162.1	1e-163	99	A3-R13/Shannon	1
*Psathyromyia* sp.	*Tamandua tetradactyla*	KT818552.1	5e-162	99	A4-R18/HP ground	1
*Psychodopygus amazonensis*	*Tamandua tetradactyla*	KT818552.1	1e-95	99	A5-R59/HP tree	1
*Psychodopygus carrerai carrerai*	*Dasypus novemcinctus*	AF493838.1	5e-86	91	A1-R4/HP ground	1
	*Dasypus novemcinctus*	KF799995.1	1e-108	89	A1-R4/HP tree	1
	*Dasypus novemcinctus*	KU253494.1	6e-100	96	A5-R59/HP tree	2
	*Dasypus novemcinctus*	KU253494.1	6e-115	99		
	*Homo sapiens*	KX365160.1	6e-136	97	A5-R59/Shannon	1
*Psychodopygus davisi*	*Coendou prehensilis*	KC463874.1	8e-150	98	A1-R4/HP tree	1
	*Coendou prehensilis*	KC463878.1	2e-57	95	A1-R4/HP ground	1
	*Dasyprocta fuliginosa*	AF437784.1	1e-107	96	A4-R18/HP ground	1
	*Dasypus kappleri*	KT818541.1	2e-105	89	A1-R4/HP ground	1
	*Didelphis marsupialis*	KJ129895.1	2e-32	97	A3-R13/HP tree	1
	*Marmosops noctivagus*	KT437714.1	5e-16	77	A3-R13/HP ground	1
	*Pecari tajacu*	JN632683.1	7e-116	91	A5-R59/Shannon	1
	*Tamandua tetradactyla*	KT818552.1	7e-42	91	A3-R13/Shannon	1
*Psychodopygus hirsutus hirsutus*	*Bos taurus*	KT343749.1	2e-33	92	A3- R13/HP tree	1
*Psychodopygus llanosmartinsi*	*Homo sapiens*	KP126162.1	5e-142	96	A5-R59/HP tree	1
*Psychodopygus* sp.	*Homo sapiens*	KX690094.1	3e-45	97	A5-R59/HP tree	1
*Sciopemyia servulolimai*	*Sus scrofa*	KJ652503.1	1e-157	99	A3-R13/HP ground	1
*Sciopemyia sordellii*	*Homo sapiens*	KP126161.1	8e-155	97	A7-R74/HP ground	1
*Trichophoromyia* sp.	*Homo sapiens*	KX697544.1	2e-166	100	A3-R13/HP tree	1
	*Homo sapiens*	DQ489515.1	6e-18	92	A3-R13/HP ground	1
*Trichopygomyia* sp.	*Choloepus didactylus*	KR336792.1	1e-128	93	A5-R59/HP ground	1
Total	* *					39

R: *Ramal* (defined by the distance in kilometres along the Trans Pacific Federal Highway BR 317)*.*

Among the 96 females analysed for the presence of *Leishmania* kDNA in the blood meal, to assess infection at the food source, 9.4% (nine/96) showed positive results in agarose gels and were subjected to the identification of the parasite species using the *hsp70* target of *Leishmania.* The analysis of the amplified sequences revealed samples with 100% identity to *L. (V.) shawi, L. (V.) braziliensis* and *L. (V.) guyanensis* (Table VII). The sample harbouring *L. (V.) guyanensis* was a female of *Sc. sordellii* that had fed on *H. sapiens* and was captured in light trap near the ground in area A7-R74. *Leishmania (V.) shawi* and *L. (V.) braziliensis* were identified in *Pa. aragaoi* and *Psa-thyromyia sp*., respectively; the *cyt b* analysis was not able to identify the food source in either sample.

The presence of *Endotrypanum* parasites was confirmed in females of *Ny. umbratilis*, *Pa. aragaoi* and *Ps. davisi*. For the last species, correlation with the probable mammalian host was only possible in the blood supply of one of the females, captured in light trap at the top of tree in area A1-R4, where the arboreal species *C. prehensilis* was identified (Table VII).

**TABLE VII:** Correlation of sandfly species with the blood gut-content and parasite species identified in the blood meal of engorged females

Species	Blood meal (*cyt b*)	Area-R/trap	Parasite species (*hsp70*)	BLAST analysis		Total
				E-value	Identity (%)	
*Lutzomyia* sp.	Low-quality sequences	A1-R4/HP ground	*L. (V.) braziliensis*	9e-42	89	1
*Nyssomyia umbratilis*	NI	A4-R18/HP ground	*Endotrypanum*	5e-80	98	1
		A4-R18/Shannon	No identity	-	-	1
*Psathyromyia aragaoi*	NI	A3-R13/HP ground	*L. (V.) shawi*	2e-68	100	1
	*Homo sapiens*	A3-R13/Shannon	*Endotrypanum*	2e-83	99	1
*Psathyromyia* sp.	NI	A3-R13/HP tree	*L. (V.) braziliensis*	2e-68	100	1
*Psychodopygus davisi*	*Coendou prehensilis*	A1-R4/HP tree	*Endotrypanum*	2e-83	99	1
	NI	A1-R4/Shannon	*Endotrypanum*	2e-83	99	1
*Sciopemyia sordellii*	*Homo sapiens*	A7-R74/HP ground	*L. (V.) guyanensis*	2e-68	100	1
Total						9

NI: not identified; R: *Ramal* (defined by the distance in kilometres along the Trans Pacific Federal Highway BR 317)*.*

## DISCUSSION

In the present study, of the 61 sandfly species captured in Brasiléia municipality, bordering Bolivia, five of them were recorded for the first time in the state of Acre: *Bi. olmeca nociva*, *Br. galindoi*, *Br. mangabeirai*, *Ps. dorlinsis* and *Th. clitella*. In addition, this investigation provides the first report of *Ps. dorlinsis* in the country, despite one solely male individual has been captured in light trap on top of tree in area A5-R59 of the forest environment. Of a total of 4,473 captured specimens, the dominance of species from *Nyssomyia* and *Psychodopygus* genera (76.4% of the captures) is consistent with previous studies carried out in Acre.^7^ ^,^ ^24^ ^-^ ^27^ Both the genera are the most medically important in the New World, represent the most abundant species and concentrate several vector species of *Leishmania*, including the most important vectors incriminated in the transmission cycle of CL in the Amazon Region.^3^ ^,^ ^24^ ^,^ ^28^

Here, the most frequent species were *Ny. shawi* (26.82%), *Ps. davisi* (12.11%) and *Ps. carrerai* (6.55%). In relation to the last two species, our data corroborate prior studies in three municipalities of the state of Acre, where these were found at frequencies of 13.35 and 6.53%, respectively.^24^ The genus *Psychodopygus* was the most dominant in Rio Branco, being *Ps*. *carrerai* and *Ps*. *davisi* the most abundant species.^29^ Although *Ps. davisi* is often being found in peridomicile ecotopes, suggesting its adaptation to anthropic environments, in our study this species has not been captured in the peridomicile areas using light traps positioned near the ground. In Assis Brasil, *Ps*. *davisi* was captured at a frequency of 21.07% and *Ps*. *carrerai carrerai* represented 0.63% of the total specimens.^26^

Relative to the highest abundance of *Ny. shawi* in our investigation, Teles et al.^26^ showed this species representing only 2.7% of the sandflies captured in Assis Brasil, and authors discuss the potential of *Ny*. *shawi* as a vector of *Leishmania* parasites in this locality, which forms a three-border area with Peru and Bolivia. Azevedo et al.^24^ identified *Ny. shawi* even less frequently (0.85%) in Acre state, and in Rio Branco its frequency was even lower (0.28%).^29^ Brilhante et al.^27^ reported the species *Ps. carrerai carrerai* (42%), *Ny. shawi* (36%) and *Ps. davisi* (13%) as the most abundant in Xapuri. In our work, these three species were also the most abundant in Shannon traps; despite the use of light attraction, the human presence during capture may bias the attraction of these anthropophilic species, considering that all three are implicated as vectors of *Leishmania* spp. (*Ny. shawi* and *Ps. carrerai carrerai* act as potential vectors in Bolivia, and *Ps. davisi* as a potential vector in the Amazon Region of Brazil).^27^ ^,^ ^30^ ^-^ ^33^

The absolute abundance of *Ny. shawi* and *Ps. davisi* (1,200 and 542 individuals, respectively) in our study, in which these species cohabit the forested ecotope, suggests their important roles in the transmission of CL agents in Brasiléia. It is important to mention that the high frequency of specimens of both *Nyssomyia* and *Psychodopygus* genera occur in the dense forest environments (45.91 and 31.12%, respectively), with lower frequencies in the peridomicile (23.81 and 1.19%, respectively); despite *Nyssomyia* genus be represented as the second more frequent in the peridomicile captures after the most dominant *Evandromyia* (29.76%). The peridomicile areas, with only 84 specimens, present lower species richness, lower diversity (except for area A2-R5), with higher indexes of equitability, and two of three areas (A6-R69 and A7-R74) exhibit high species dominance (*Ev. saulensis* and *Th*. *auraensis*, respectively). The dense forest areas, performing 98% of the total of captures, present higher species richness, higher diversity (being area A4-R18 the one with the highest diversity index), with lower index of equitability (except of area A4-R18 with equitability similar to that of sampled areas in the peridomicile), and high species dominance could only be observed in area A1-R4 (*Ny*. *shawi*). The overall higher abundance and species richness in the forest environment were expected, if one considers the higher frequency of sandflies in non-degraded environments such as the high-density forest, in association with a greater sampling effort applied in these forested areas where the three methodologies of capture were used, compared to the peridomicile areas (only light traps on the ground). The captures with Shannon revealed the highest sandflies average followed by light traps positioned on the top of trees (84.1 and 28.3, respectively), being *Ny*. *shawi* the most captured species in these traps, thus demonstrating a dominant behaviour in the dense forest environment and indicating the anthropophilic behaviour of this species.

Despite the human occupation and intense deforestation in Brasiléia, these areas of remaining forest suffer less anthropic impacts than that of the peridomicile environment, and consequently contribute in maintaining the higher richness on species distribution. Taking into account the original wild behaviour of sandflies, the distribution and species abundance are directly influenced by forested areas, as stated in a study on sandfly faunal diversity in southern Brazil, where a correlation between insects’ richness and the existence of remaining forests was observed.^34^ As previously reported and corroborating with the data here obtained, high species diversity is frequently found in the Amazon Region.^33^ ^,^ ^35^ ^,^ ^36^

Studies on *Leishmania* infection/detection in sandflies contribute with functional indicators of the parasite transmission intensity in endemic areas. In this context, the search for *Leishmania* DNA in the sandfly fauna of Brasiléia could suggests the implication of positive species as potential vectors of CL agents in the sampled areas, being that the first essential criterion to be regarded as suspected vectors.^37^ Here, we found 25 positive specimens out of 864 females individually analysed in the evaluation of possible natural infections, corresponding to a detection rate of 2.9% for *Leishmania* spp. The positive specimens were trapped in all areas of forest environment and only in one area of peridomicile (A2-R5). The minor sampling efforts for the peridomicile environment resulted in few specimens captured and a minor chance of finding positive sandflies. Our results revealed the circulation of *L. (V.) braziliensis* and *L. (V.) guyanensis* in Brasiléia, the second municipality in the state of Acre with the highest number of notifications of human cases of CL; in the period 2012 to 2017, the Rio Branco and Brasiléia notified 2,652 and 2,025 cases, respectively. 

Relative to the higher number of specimens submitted to *Leishmania* DNA-detection, *Ps. davisi* represented the species with the highest positivity (seven/70, 10%) for *L. (V.) braziliensis*. All positive specimens were captured in the forest environment, six of them in Shannon and one in light trap positioned on the ground. Other studies in the Amazon Region point to *Ps. davisi* as a putative vector of *L. (V.) braziliensis* and *L. (V.) naiffi* in the states of Rondônia^35^ ^,^ ^38^ and Pará,^39^ respectively. Despite its wild habitat, as observed in the present work and also in Rio Branco,^29^ ^)^ *Ps. davisi* has been frequently found in peridomestic environments associated to the occurrence of CL, suggesting its adaptation to anthropic environments.^29^ ^,^ ^40^ In the municipality of Assis Brasil, this species was found to be DNA positive for both *L. (V.) braziliensis* and *L. (V.) guyanensis.* ^26^ In Rio Branco, *Ps*. *davisi* was one of the most abundant species and *L*. (*V*.) *braziliensis* was identified in one solely individual.^29^ Considering its anthropophilic behavior, high density and possible infection with *Leishmania* parasites, *Ps. davisi* is incriminated in the enzootic and zoonotic cycles of CL in the Amazon Region.^33^ ^,^ ^35^ ^,^ ^39^ ^,^ ^41^ Our blood gut-content analysis also supports the involvement of this sandfly species in the enzootic transmission cycle in Brasiléia. 

The species *Ny. shawi* is considered anthropophilic^42^ and is involved in the CL cycle transmission in Bolivia, where it has been found to be positive for *L. (V.) braziliensis* and *L. (V.) guyanensis*,^43^ whereas until now its potential to act as vector of CL agents in Brazil still needs confirmation. Similar to the findings of García et al.^43^ in Brasiléia, which shares a border area with the municipality of Cobija in Bolivia, DNA of both *L. (V.) brazi-liensis* and *L. (V.) guyanensis* parasites was identified in *Ny*. *shawi* (four/180, 2.2% total positivity), captured in the dense forest environment in light traps on the ground and Shannon. These two parasites were also identified by our group in cutaneous lesions of individuals living in Brasiléia.^44^ According to the essential criteria of Killick-Kendrick,^45^ these results provide further elements on the putative vector role of *Ny. shawi* regarding *L. (V.) brazi-liensis* and *L. (V.) guyanensis* enzootics in the study area. In the blood gut-content analysis, *Homo sapiens* DNA was identified in this sandfly species, providing support for its anthropophilia in the region. Recently, flagellates were observed in the mid and hind portions of the gut in *Ny. shawi* captured in Xapuri,^27^ a municipality of Acre bordering Brasiléia, as well as in Assis Brasil, another neighbouring municipality, leading to a discussion about the potential vector role of *Ny. shawi* in the three-border area of Brazil, Bolivia and Peru.^26^

*Ny. umbratilis* is the main vector of *L. (V.) guyanensis* and is considered a fairly anthropophilic species.^46^ A study carried out in Peixoto de Azevedo, in Mato Grosso state, showed a possible association of *Ny. umbratilis* with the transmission of *L. (V.) braziliensis,* ^47^ reinforcing our results regarding the identification of this parasite in one specimen of *Ny. umbratilis* (one/44, 2.27%) captured in light trap on top of tree in area A3-R13 of forest environment*.*

A single specimen of *Ny. antunesi* tested positive for *L. (V.) braziliensis* DNA (one/19, 5.26%) and was captured in light trap on the ground in the peridomicile (area A2-R5). This species has been associated with wild animals and peridomiciliary environments^48^ and also occurs in Peru and Bolivia.^13^ A previous study in Acrelândia (state of Acre) reported this species in a high frequency (59.1%) in the peridomicile environment and forests edges;^7^ in Rio Branco, *Ny. antunesi* was found in low abundance in forest environments.^29^ This species has been associated with foci of visceral^49^ and CL,^50^ being also regarded as a suspected vector of *L. (V.) lindenbergi* in the state of Pará.^48^

One solely *Ps. hirsutus hirsutus* captured in Shannon in the forest environment (area A4-R18) was found positive for *L. (V.) braziliensis* (one/17, 5.9%). Other works show this species as a potential vector of *L. (V.) braziliensis* and *L. (V.) naiffi* in the Amazon Region.^24^ ^,^ ^35^ ^,^ ^46^ In the municipality of Xapuri, *Ps. hirsutus hirsutus* was recently identified as having been parasitised by flagellated forms.^27^

For the other species with positive results on the search of *Leishmania* DNA *(Ev*. *termitophila*, *Pintomyia* sp., *Pi. nevesi* and *Brumptomyia* sp.), the high detection rates corresponded to a lower number of captured individuals (or few specimens submitted to molecular diagnosis) and with only one positive sample (Tables IV and V). It is important to highlight the first report of the finding of *L.* (*V*.) *braziliensis* DNA in *Ev*. *termitophila* and *Brumptomyia* sp. in the state of Acre; both sandflies were captured in light traps on the ground in the A2-R5 peridomicile area. Recently, DNA of this parasite was also found in *Pi. nevesi* captured in Rio Branco.^29^ In eight phlebotomine species it was not possible to infer the identity of the species of *Leishmania* (*Ny*. *shawi*, *Nyssomyia* sp., *Ny*. *whitmani*, *Pintomyia* sp., *Ps*. *amazonensis*, *Psychodopygus* sp. and two samples of *Ps*. *davisi*). 

The relatively low identity value of 89% obtained for *L*. (*V*.) *braziliensis* DNA in one specimen from each of *Ny*. *umbratilis*, *Pi*. *nevesi* and *Ps*. *davisi* may reflect the high intraspecific variability found for the “braziliensis complex” in Acre.^51^ In two species of sandflies, *Ny*. *shawi* and *Psychodopygus* sp., it was possible to confirm 100% identity for *L*. (*V*.) *guyanensis*. Despite the high abundance of *Ps*. *carrerai carrerai*, for which 293 specimens were captured in all areas of forest environment, we did not find any positive sample for the presence of *Leishmania* DNA. Similar results were obtained in Rio Branco, where this species, together with *Ps*. *davisi*, were the most abundant and a positive result for *L*. *(V.) braziliensis* detection was only observed in one specimen of *Ps*. *davisi.* ^29^

The state of Acre in the Amazon Region has an extensive biological representativeness, comprising almost 40% of the mammal’s species in Brazil and 4.5% worldwide. Several of these wild animals in Acre could represent a permanent food supply for sandflies.^52^ By studying the blood gut-content of these insects, it is possible to infer possible reservoirs acting in the maintenance of the enzootic cycle and better assessing the degree of anthropophilia of suspected vector species.^53^ In the present study, the blood gut-content analysis was able to properly identify 13 mammalian species in 39 sandfly samples. Interestingly, *Homo sapiens* represented the main source of feeding detected in these specimens (41.02%). Female sandflies fed on humans were captured in all areas of forest and peridomicile environments, with the exception of area A6-R69 (peridomicile) that did not present engorged females. From these, 87.5% were captured in the dense forest areas, corroborating with the human local activities in the wild environment for the extraction of wood, oils, fruits, rubber and hunting. More than half of the sandfly species whose blood gut-contents were identified, included at least one specimen that had fed on human blood. For example, in *Ny. shawi*, *Nyssomyia* sp., *Trichophoromyia* sp. and *Ev. saulensis*, more than one individual that had fed on humans was identified. As discussed previously, *Ny. shawi* is considered anthropophilic^42^ and probably affects the transmission of *L. (V.) braziliensis* and *L. (V.) guyanensis* in Bolivia.^43^ In Rio Branco, *Ev*. *saulensis* was the most abundant species in the captures using Shannon and with dominance in the forest environment, confirming its anthropophilic behavior.^29^ Other studies also highlight the role of *Ev. saulensis* and *Trichophoromyia* sp. as possible vectors in the state of Acre.^26^ ^,^ ^29^ ^,^ ^54^ As recently discussed, the putative vector importance of some species of the *Tricho-phoromyia* genus (including those found in Acre) seems to be increasing,^28^ but as far as we know our results are the first to demonstrate the man-biting behavior of specimens of this genus. As mentioned before, the females identified as *Trichophoromyia* probably belong to *Th*. *octavioi* and *Th*. *auraenis*, since up to now they are not distinguishable. *Ps. carrerai carrerai* and *Ps. llanosmartinsi,* which were also found to have fed on humans in our work, have been confirmed as vectors of *L. (V.) braziliensis* in Bolivia.^30^ ^,^ ^32^

Among the wild animals identified in the blood supply samples, armadillos were the most frequent and were represented by two species, *D. novemcinctus* and *D. kappleri*, which were exclusively identified in females captured in the dense forest areas A1-R4 and A5-R59. The former was identified only in *Ps. carrerai carrerai* that showed more selective feeding habits, exhibiting four specimens fed on this same species of armadillo. Although more than a half of the engorged *Ps. carrerai carrerai* females have been captured in light traps at the top of trees, this sandfly is commonly found in resting sites with fallen leaves on the ground.^55^ A comprehensive discussion on the vertical/horizontal dissociation between feeding and resting sites can be found.^56^ These authors report the capture of *Ps. hirsutus hirsutus* in trees canopy, including an infected one, and this might be interpreted as circumstantial evidence of a *L*. *(V.) naiffi* arboreal enzootic cycle involving other reservoirs in the Tapajós National Forest, Belterra municipality in the state of Acre. Nonetheless, *L*. *(V.) naiffi*’s accepted reservoir is the nine-banded armadillo *D. novemcinctus*, an obligatory terrestrial mammal;^57^ although *L. (V.) guyanensis* has also been detected in this and in other armadillo species.^55^ On the other hand, it could just be a natural vertical migration of flies that became infected on the ground. An example of this is the vertical migration of *Ny. umbratilis* that becomes infected from the arboreal two-toed sloth (*C. didactylus*), but transmission to man occurs at ground level when the fly descends from the canopy during the day.^57^ In our study, we identify one specimen of *Ps*. *hirsutus hirsutus* fed on *B. taurus* and captured on the top of tree, possible reflecting another example of natural vertical migration. 

Samples identified as *T. tetradactyla* were the second most common food source among wild animals and sandflies fed on this species (*Ps. davisi*, *Ps. amazonensis*, *Psathyromyia* sp. and *Evandromyia* sp.) were captured in all traps in the dense forest environment, their natural habitat, in areas A3-R13, A4-R18 and A5-R59 and also in light trap positioned on the ground at the edge of the forest in area A2-R5 (peridomicile). The finding of *Ps. amazonensis* feeding on *T. tetradactyla* is curious, because this sandfly is likely to feed on armadillos (*D*. *kappleri*, *D*. *novemcinctus*).^58^ *T. tetradactyla* is currently the only species of anteater from which *Leishmania* was isolated, in which *L. (V.) guyanensis* and *L*. *(L.) amazonensis* were isolated in Brazil and Ecuador, respectively.^55^

In our study, other species of wild animals also played important roles in the ecoepidemiology of leishmaniasis and the maintenance of sandfly species. The order Marsupialia was represented by the species *M. noctivagus* and *D. marsupialis*, which were identified in the blood gut-content in two specimens of *Ps. davisi* captured in area A3-R13 of the dense forest environment. *Didelphis* is the genus with the greatest dispersion on the continent, and because of its synanthropic nature, it is one of the most investigated mammals relative to infection by *Leishmania* spp.^55^ *D. marsupialis* has been identified as being naturally infected by *L. (L.) infantum*,^59^ *L. (V.) braziliensis*,^60^ *L. (L.) amazonensis* ^61^ and *L. panamensis.* ^62^ In relation to rodents, several works have identified different species infected by *L*. (*L*.) *amazonensis*, including species of *Dasyprocta.* ^55^ ^,^ ^60^ ^,^ ^62^ In the present work, this genus was represented by the species *D. fuliginosa*. Another rodent identified here, *A. paca*, is the only wild species that was previously found to be infected by *L*. *(V.) lainsoni* in the state of Pará.^63^ Different species of *Leishmania* have been detected in *C. didactylus*. For example, this species of sloth is considered a potential reservoir of *L*. (*V*.) *guyanensis*,^60^ ^,^ ^64^ and in Brazil, infection by *L*. *(V.) shawi* has also been reported.^46^ Representatives of Artiodactyla were also found in our work. The identification of sandfly females that had fed on domestic pigs (*S. scrofa*) and oxen (*B. taurus*), exclusively in area A3-R13, corroborates the local characteristics of this site, which was the only area of forest environment where pigs occurred in the peridomicile and with the presence of an ox-breeding site close to the capture area.

Engorged sandfly females subjected to the analysis of blood gut-content showed positive results for the presence of *Leishmania* DNA. Sequencing results revealed samples with 100% identity to *L. (V.) shawi*, *L. (V.) braziliensis* and *L. (V.) guyanensis* from *Pa. aragaoi*, *Psathyromyia* sp. and *Sc.* sordellii, respectively. Due to the blood gut-contents of these sandflies, we cannot associate them with the transmission of *Leishmania* in the studied areas. Beyond this, it cannot be ascertained that the tested blood and *Leishmania* have been ingested in the same occasion. A second blood meal after *Leishmania* infection (from another blood) is also possible. These results suggest that infection could be an occasional finding and that these insects have had previous contact with potential reservoirs of these species of *Leishmania* in the sampling areas (A1-R4 for *L*. *braziliensis*, A3-R13 for *L*. *braziliensis* and *L*. *shawi*, and the peridomicile area A7-R74 for *L*. *guyanensis*). The presence of the parasite *Endotrypanum* was identified in females of *Ny. umbratilis, Pa. aragaoi* and *Ps. davisi*. Relative to the last, correlation with the probable mammalian host was possible in only one of the females, in which the species *Coendou prehensilis* was identified in area A1-R4 (light trap on top of tree). *Pa. aragaoi* is recognised as showing preference for feeding on dasipodids such as armadillos (known to harbour *Leishmania* species, parasites of the *Trypanosoma* genus and *Endotrypanum*) and uses the refuges of these animals as resting places.^55^ ^,^ ^62^ Here, the specimen of *Pa*. *aragaoi* presenting *Endotrypanum* DNA was captured in area A3-R13 (Shannon) and human blood was identified in its gut-content; probably, this female was already parasitised by *Endotrypanum* spp. and had performed a recent blood meal on humans. The identification of *Endotrypanum* spp. parasites is noteworthy; these parasites only occurred in samples of engorged sandflies, which has not previously been seen by our group in non-engorged females subjected to the diagnosis of *Leishmania* DNA-detection. *Endotrypanum* spp. belong to the Trypanosomatida order and are very closely related to *Leishmania*, being currently classified in the Paraleishmania section, which allowed their detection in the target kDNA analysis.^65^

This finding demonstrates the need to use complementary techniques, for instance hybridisation with specific probes and/or PCR targeting the hsp70 *Leishmania* gene, to accurately confirm the species or subgenera involved. By using this marker followed by sequencing the amplicons, we were able to demonstrate the presence of *L. (V.) braziliensis*, *L. (V.) shawi* and *L. (V.) guyanensis*, which are etiological agents of CL, circulating in the study area among sandflies and possible hosts/reservoirs. These data are in agreement with previous analyses including CL patients in the state of Acre, in which highly diverse *Leishmania* species were isolated from clinical samples, including *L. (V.) braziliensis*, *L. (V.) shawi*, *L. (V.) guyanensis*, *L. (V.) lainsoni*, as well as possible mixed infections by *L*. (*V*.) *guyanensis*/*L*. (*L*.) *amazonensis* and *L*. (*V*.) *naiffi*/*L*. (*V*.) *lainsoni*.^51^ ^,^ ^54^ ^,^ ^66^ These authors reinforced the hypothesis that the transmission of these parasites occurs with a higher frequency in rural/forest environments.

Overall, the data obtained in this study are highly relevant, bearing in mind that little is known about the agents, vectors and reservoirs that constitute the CL epidemiological chain in the state of Acre, a region of Brazil with high risk of contracting the disease by the human population.
